# Variation in CD4 cell count among IDUs versus sexually-infected HIV-positive naïve patients in Romania

**DOI:** 10.1186/1471-2334-14-S4-O11

**Published:** 2014-05-29

**Authors:** Iulia Niculescu, Eliza Manea, Simona Paraschiv, Ionelia Bâțan, Adrian Abagiu, Adriana Hristea, Raluca Jipa, Cătălin Tilişcan, Raluca Mihăilescu, Adrian Streinu-Cercel, Victoria Aramă, Dan Oțelea

**Affiliations:** 1 Clinical Department, National Institute for Infectious Diseases “Prof. Dr. Matei Balş”, Bucharest, Romania; 2 Molecular Diagnostics Laboratory, National Institute for Infectious Diseases “Prof. Dr. Matei Balş”, Bucharest, Romania; 3 Carol Davila University of Medicine and Pharmacy, Bucharest, Romania

## 

Since 2011 Romania is experiencing a dramatic increase of newly diagnosed HIV infections among injecting drug users (IDUs), mainly linked to the introduction of new psychoactive substances (NPS) on the Romanian market, their use being related to higher levels of risk behavior compared to opioid abuse. There is no sufficient data showing the natural course of HIV in subjects infected through IDU – the majority are ART-naïve due to recently acquired infection (many are asymptomatic and have a CD4 cell count over 350 cells/cmm) and poor adherence. Our objective was to determine if IDUs have a faster decline in CD4 cells than sexually-infected patients.

We performed a retrospective study, including ART-naïve HIV-positive patients diagnosed between January 2011 and June 2013 at the National Institute for Infectious Diseases “Prof. Dr. Matei Balş”, who had 2 subsequent CD4 cell count determinations over a timespan of 6-24 months and a baseline CD4 count higher than 350 cells/cmm. Among 1,248 newly diagnosed patients, 234 met these inclusion criteria. The data were statistically analyzed using SPSS version 17 (independent sample t-test, Mann-Whitney test, linear regression; the significance level was set at 0.05).

The majority (80%, 187/234) were men and the median age was 29 years (15-76). More than half of the patients (138; 59%) were former or active IDUs, with low socioeconomic status, most of them injecting both opioids and NPS and 55 (40%) of them were in detention at the moment of HIV diagnosis. Among them, 98% were coinfected with HCV, as opposed to only 21% of the sexually-infected patients. Subtype analysis was performed for 64 patients and revealed the following subtypes and circulating recombinant forms (CRF): 50 F1, 3 B, 10 CRF14_BG and 1 CRF14_F. There was no significant difference of CD4 cell count between the two groups at baseline (p=0.55). The median variation of CD4 cells in IDUs was 150 and 42 in the non IDUs group. IDUs had a statistically significant CD4 cell decline (p=0.01). HCV coinfection was also correlated with a faster decline in CD4 cells (p <0.001).

The more rapid decline of CD4 cells among IDUs could be explained by direct effect of drugs, differences in the virulence of the HIV strains circulating among IDUs and HCV coinfection. These data highlight the importance of carefully monitoring IDUs with HIV infection and of initiating ART earlier in this risk group.

